# A new look at the theoretical causes of endometriosis: Narrative review

**DOI:** 10.18502/ijrm.v22i5.16433

**Published:** 2024-07-08

**Authors:** Abdelmonem Awad Hegazy

**Affiliations:** ^1^Department of Basic Medical Sciences, Faculty of Dentistry, Zarqa University, Zarqa City, Jordan.; ^2^Department of Human Anatomy and Embryology, Faculty of Medicine, Zagazig University, Zagazig City, Egypt.

**Keywords:** Sexual behavior, Endometrioma, Menstruation, Retrograde, Painful, Fertility.

## Abstract

Endometriosis is a major health concern in women who have it. Unfortunately, there is no definitive cure except panhysterectomy with its sequelae including induction of premature menopause due to loss of ovaries. Therefore, revealing the causes of this puzzling disease is necessary to avoid contracting it, and to spare women the health disorders resulting from it and the difficulties of treating it. We aimed to study endometriosis with a focus on its theoretical causes. Its classification reports and theories of pathogenesis were identified and studied from available database searches. The causes of endometriosis remain mysterious. Many theories have been proposed to explain the etiology, but retrograde menstruation (RM) remains the closest in this regard. Although this theory is the most accepted in the pathogenesis of endometriosis, its causes are still a matter of debate, especially in women who do not suffer from obstructions to menstrual outflows, such as cases of congenital cervical stenosis and imperforate hymen. It is suggested in some studies that there may be a relationship between women who engage in sexual activity during menstruation and the development of endometriosis. It is concluded that endometriosis is a painful and debilitating disease. Identifying its causes is essential to control the disease and avoid any burdens on health. RM is the main theory for its pathogenesis but its causes are still uncertain. Sexual activity during menstruation may be a possible cause of RM but needs more evidence. Future studies are recommended to reveal all aspects of the pathogenesis of endometriosis.

## 1. Introduction

Endometriosis is a benign and chronic estrogen-dependent inflammatory disease that affects approximately 10% of women of childbearing age, increases up to 50% in infertile women, and up to 60% in patients with chronic pelvic pain (1). Although endometriosis is mostly benign, it can develop into ovarian cancer (2). It represents the third leading cause of hospitalization for women in the United States and one of the leading reasons for hysterectomy (3). Although the disease seems to be a product of modern life or a disease of the 21 ${\;}^{\text{st}}$ century, the first references and disorders associated with endometriosis were mentioned in ancient Egypt in 1500 BC (4).

Endometriosis arises from the endometrium, which is the anatomical and functional inner layer of the uterus (5). The endometrium represents the inner layer of the 3 layers that make up the structure of the uterine wall, followed by the bulky smooth muscle layer called the myometrium, and finally, the surrounding thin outer layer called the perimetrium. The endometrium, which is also called the intracavitary mucous lining of the uterus, serves to accommodate the implantation of the developing embryo as well as to prevent the adhesion of the opposite layers of the myometrium to each other and thus preserve the uterine cavity. The endometrium exhibited 3 layers in structure. Its superficial layers undergo shedding in each menstrual cycle, leaving only the basal layer, which rebuilds the endometrium again during each cycle (6). This cycle is controlled by the hormones estrogen and progesterone secreted by the ovaries. However, the endometrium after menopause and in the absence of hormonal control, loses cyclical changes undergoing atrophy and diminishes to ≤ 5 mm in thickness (7). In this review, we have tried to clarify the theories and possible causes, focusing on female sexual activity during menstruation (SADM) as a possible avoidable cause. Identifying the possible causes of endometriosis is essential to control the disease and avoid its health burdens. With proper knowledge of the causes, its occurrence can be greatly reduced and can be avoided as much as possible.

## 2. Ectopic endometrium

The presence of endometrial glands and stroma outside of their normal location in the lining of the uterus is called ectopic endometrium (8). The ectopic endometrium occurs in 2 main forms either through expansion deep into the wall of the uterus or extending far outside the uterus. Deep extension of the endometrial tissue into the myometrium caused by hyperplasia of the basal layer leads to adenomyosis in which the uterus becomes homogenous, enlarged, and spherical with complaints of dysmenorrhea and menorrhagia (9). If the extension is in a localized form, the condition is called adenomyoma (10). Treatment of such cases includes medical and surgical procedures. Medical treatment includes hormonal therapy such as estrogen and progestin pills as well as nonsteroidal anti-inflammatory drugs, while the surgical procedure is hysterectomy which is the definitive treatment (9). On the other hand, the implantation of endometrial tissue outside the uterus is called endometriosis.

## 3. Endometriosis

**Figure 1:**
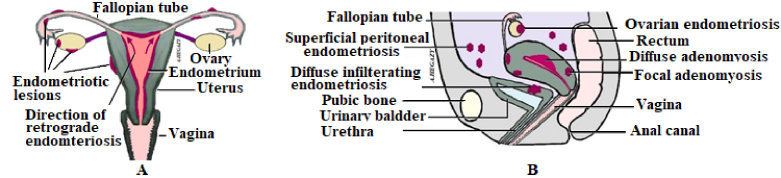
A) Female genitalia with RM and endometriotic lesions, B) Sagittal section of female pelvis and genitalia with adenomyosis and endometriosis lesions.

Endometriosis often affects the pelvic viscera including the fallopian tubes, ovaries, urinary bladder, intestines, and peritoneum (Figure 1). However, it rarely affects other structures outside the pelvis such as the abdominal wall and diaphragm, and in rare cases, it may extend to the pleura and nerve tissue (11). Endometriosis represents a challenging gynecological disease with no conclusive diagnosis except for the use of laparoscopy. Diagnosis is based on clinical suspicion depending on patients' complaints, signs, and magnetic resonance imaging but is confirmed by laparoscopy (12). There is a significant impact of endometriosis in women in terms of gynecological morbidity and economic burden (13). Moreover, the number of visits to hospitals for treatment of endometriosis is still gradually increasing (14). Endometriosis increases the economic burden either directly on health care or indirectly through lost productivity over the years. It was estimated at $22 billion in 2002 and $69.4 billion in 2009 in the United States (15).

**Figure 2:**
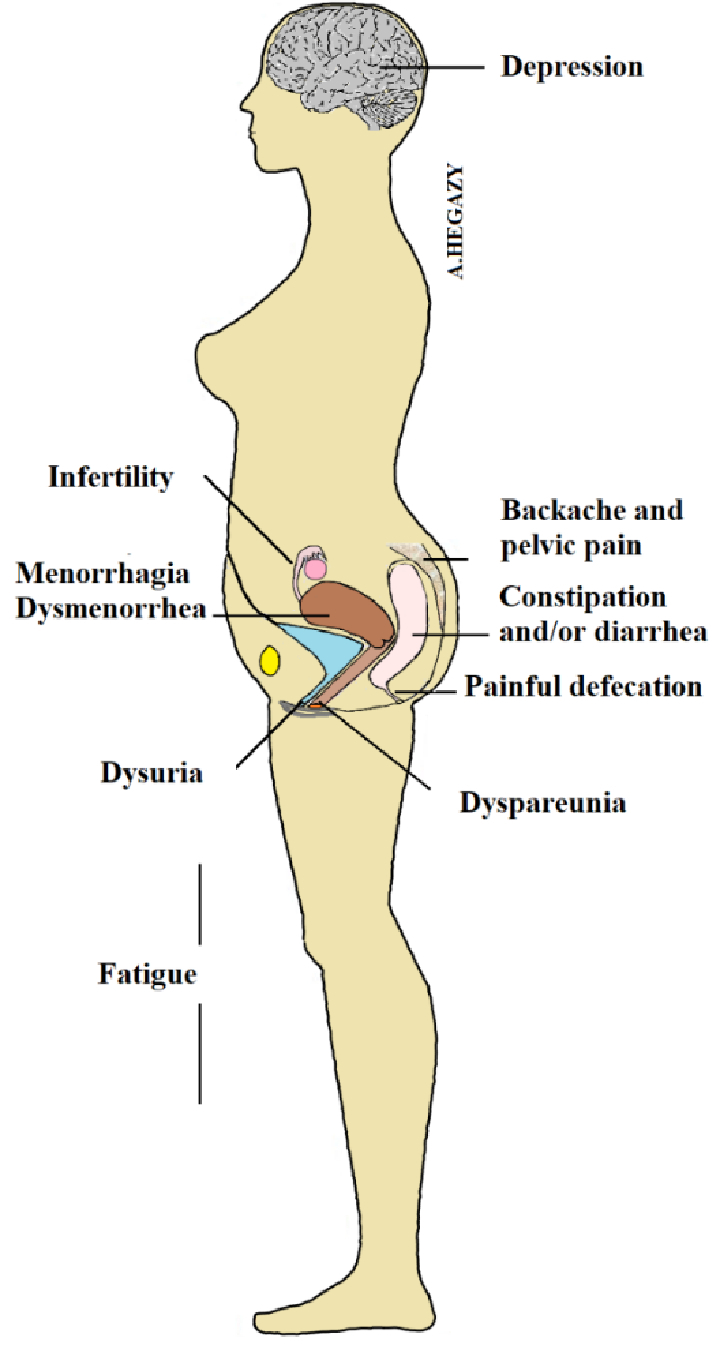
Some symptoms of endometriosis.

This disease is a painful and debilitating disorder, often associated with numerous symptoms including chronic pelvic pain, back pain, bowel movement disturbances, dysuria, dyspareunia, menorrhagia, and dysmenorrhea as well as pelvic adhesions and impaired fertility (Figure 2) (16). Moreover, it may also lead to adverse pregnancy outcomes including placenta previa, placental abruption, gestational diabetes, hypertensive disorders, premature labor, stillbirth, and postpartum hemorrhage (17). Endometriosis negatively affects a woman's daily life regarding almost all activities including sleep, emotions, social interaction, work, finances, sex life, and fertility (18).

The pathogenesis of endometriosis-related disorders is assumed to be due to periodic bleeding in ectopic foci of the endometrium. This can lead to chronic inflammations and adhesions in the pelvis along with debilitating chronic pain, dysuria, dyskinesia, and infertility (19, 20). It is also hypothesized that ectopic endometrium causes an immune response and increases the inflammatory reaction through the production of cytokines. This then leads to the elevation of angiogenic factors and interleukins, which facilitate further pelvic adhesions and angiogenesis as well as further spread of endometriotic lesions (17).

There is no definitive treatment for endometriosis. All measures, whether medical, surgical, or both, aim to relieve symptoms so as not to interfere with a normal lifestyle. Unfortunately, the only definitive treatment for endometriosis can be through a panhysterectomy with the removal of the entire uterus, tubes, and ovaries (21). This type of treatment, which involves the removal of both ovaries, leads to early menopause with complications affecting young women (22). Therefore, it is necessary to know its pathogenic causes to avoid its occurrence as much as possible. Therefore, understanding its causes and pathogenesis may help avoid its occurrence and thus eliminate its consequences (12).

Endometriosis affects women of childbearing age and recedes with the onset of menopause. It is an aggressive and recurrent disease and recedes with the onset of menopause (13). It most commonly occurs between the ages of 25 and 35 yr. Moreover, it should not be excluded when diagnosing adolescent girls who complain of chronic pelvic pain. There are many theories to explain the pathogenesis of endometriosis but none of them has been confirmed yet. Proposed theories to explain its causes include the theory of regurgitation or retrograde menstruation (RM), the metastatic theory meaning the distant spread of endometrial cells or tissues through blood vessels or lymphatic vessels to other parts of the human body, the metaplastic theory with transformation of peritoneal mesothelium, and the stem cell theory (23, 24). Among these causes, RM remains the most compatible etiological theory for endometriosis. Endometriosis of RM has recently been demonstrated through DNA analysis. There is convincing evidence from DNA screening that pelvic endometriosis including superficial or deep endometrial infiltration, and ovarian endometriosis share similar or identical genetic makeup as the mucosa within the uterine cavity (5).

Some authors have reported that endometrial reflux occurs in approximately 76–90% of women depending on the patency of the fallopian tubes, but without further development of endometriosis (DOE) (25). However, the diagnosis of endometriosis may not be definitive without surgery. Therefore, it can only be diagnosed by laparoscopy or laparotomy. These procedures are not usually performed routinely on any woman but are performed when needed, such as in cases of tubal infertility and a history of pelvic inflammatory disease (26). Therefore, we believe that the previously mentioned high rate of up to 90% of endometrial reflux based on fallopian tube integrity alone may not be accurate and needs to be reinvestigated. The diagnosis of the disease may be delayed (16). Other authors have found that the prevalence of RM is higher in women with endometriosis (97%) than in women without it (60%) (27).

## 4. Classification of endometriosis 

Currently, there is no gold standard classification system that represents an issue of concern in the treatment of endometriosis. This classification system is essential not only to clarify communication between clinicians but also to standardize treatment strategy (14). Endometriosis has been classified into 3 types (Figure 1). These include superficial peritoneal, ovarian, and deep infiltrating types (28, 29). Some authors added ovarian endometriomas to deep lesions, thus classifying lesions into superficial and deep endometriosis (12). The superficial peritoneal type affects 15–50% of all cases of endometriosis in which endometriotic cells are implanted in the peritoneum. This transplanted tissue goes through 3 evolutionary steps: Red, black, and white. The first red step is a highly active and vascular lesion. The second black stage is advanced endometriosis followed by the last white stage which represents quiescent or healed endometrium and is also called latent lesion (29, 30). Deep implants are defined as adenomyosis externa that infiltrate the peritoneum by ≥ 5 mm and are observed in the pouch of Douglas and the uterosacral ligaments (12, 31). There is some ambiguity regarding the pathogenesis of endometriosis, which is reflected in the lack of consensus on the classification of the disease (12). Some authors classified superficial and deep endometritis as 2 different diseases with different pathogenetic mechanisms, while others considered them to be different clinical manifestations of the same disease (12, 32).

The classification most commonly used to explain the severity of endometriosis in patients is the classification proposed and revised by the American Society for Reproductive Medicine (ASRM) known as the revised ASRM classification. In this classification, endometrial lesions in the ovaries, fallopian tubes, and peritoneum were divided into 4 stages according to the size and severity of the lesion. However, there are some criticisms of this system including the poor reproducibility of revised ASRM classification, the lack of correlation between pain severity and infertility with the proposed staging, and the presence of a discrepancy between actually diagnosed lesions and histologically diagnosed lesions (14).

## 5. Theories of endometriosis

Although there is no consensus among scientists on a theory that covers all endometriosis disorders, there are some risk factors that enhance its occurrence in some women and not others. These factors include hormonal, genetic, and immune system changes (4, 33). The main theories proposed to explain endometriosis include the following (Table I).

**Table 1:** Theories of endometriosis


**Theory **	**Proposed mechanism**
**Retrograde menstruation**	Retrograde flow of menstrual contents may allow implantation of endometrial tissue into the peritoneal cavity
**Coelomic metaplasia**	Metaplasia of the peritoneal tissues or cells to form endometrial tissue may occur in all derivatives of coelomic epithelium
**Embryonic remnants**	It is a metaplasia of the embryonic remnants such as embryonic Müllerian ducts that can differentiate into endometrial tissue
**Endometrial metastasis**	Endometrial metastasis is a theory in which small pieces of endometrial tissue can spread through the uterine lymphatic vessels or blood vessels during menstruation
**Stem cell recruitment**	Stem cells from the endometrium or bone marrow are supposed to cause endometriosis
**Immune dysfunction**	Immune dysfunction can play a role in the pathogenesis of endometriosis through angiogenesis as well as the growth and invasion of endometrial cells
**Hormones' imbalance**	An imbalance of hormones such as estrogen and progesterone may play a role in the DOE
**Environmental factors**	Some environmental factors, such as sedentary living, alcohol intake, and air pollution, are hypothesized to play a role in the DOE
DOE: Development of endometriosis	

### RM

This theory, proposed by Simpson in 1925, remains the most widely accepted (34). In this theory, the retrograde passage of menstrual blood containing sloughed endometrial tissue occurs through the open lumen of the fallopian tubes to embed itself in the peritoneal cavity and viscera (11, 12). Angiogenesis then occurs after endometrial implantation leading to the development and growth of endometriotic lesions. This theory may be supported by the discovery in such cases of activation of peritoneal macrophages responsible for the production of angiogenic factors such as vascular endothelial growth factors (11). Furthermore, patients with endometriosis have been found to have greater amounts of retrograde menstrual fluid than healthy women (12). In addition, experimental inoculation of pelvic menstrual endometrial tissue resulted in DOE in up to approximately half of the animals, and in 100% of animals after inoculation for 2 consecutive cycles (35, 36). Histological examination of these advanced lesions revealed similarities to endometriosis in humans (12, 36). Other evidence supporting this theory is the DOE in cases of congenital obstruction of the normal vaginal route for emptying menstrual contents, such as cases of imperforate hymen and cervical stenosis (37, 38). However, this theory fails to explain rare cases of endometriotic lesions in boys, prepubertal girls, and newborns (12). The extremely rare cases of male endometriosis may be explained by another theory suggesting the presence of embryonic cell remnants from Müllerian ducts in males that may develop to form the endometrium (39). For newborn females, children, and young adolescent girls, endometriosis can be attributed to RM caused by withdrawal bleeding that may occur in neonates due to the cessation of ovarian “maternal” hormones after birth (40).

### Theory of coelomic metaplasia

This theory was first proposed in 1924 by Robert Mayer. This theory supposes the presence of metaplasia of the original coelomic epithelium to form endometrial tissue. Therefore, it depends on the presence of non-uterine cells that may, under certain circumstances, undergo metaplasia to form endometriosis (41). This theory could explain some rare conditions that Sampson's previous theory could not, including endometriosis in males and also in females with uterine aplasia as in Mayer-Rokitansky-Küster-Hauser syndrome (11). In Mayer-Rokitansky-Küster-Hauser syndrome there are congenital malformations of the Müllerian ducts that clinically manifest as aplasia of the uterus and upper part of the vagina (42). It may also explain the endometrioma of the ovary as there may be a metaplasia in the coelomic epithelium that covers the ovary and then extends into the ovarian cortex. However, the factors promoting these changes have not been elucidated (11).

### Embryogenetic theory

This may be a type of metaplasia. However, it is not limited to the coelomic epithelium as in the metaplasia theory. The theory assumes the presence of embryonic remnants that can differentiate into endometrial tissue (11). This is based on the development of the female reproductive ducts from the embryonic Müllerian ducts. These ducts disappear in the male, but some remnants may remain and develop into endometrial tissue (6, 11). It is suggested that there may be some factors involved in cell differentiation and transport during embryonic development that could sustain the spread of embryonic cells (primordial endometrial cells) (11). The theory postulates the survival of residual embryonic cells in the Müllerian and/or Wolffian ducts that may develop into endometrial tissue under the influence of estrogen (43). This may add an explanation to endometriosis in males because of the presence of embryonic cells in the Wolffian ducts (11).

### Metastatic theory 

In 1927, Simpson postulated the theory of endometrial metastasis in which small pieces of endometrial tissue could be disseminated through uterine lymphatics or blood vessels during menstruation (44). This benign metastasis may explain the presence of very rare cases of endometrial lesions in lymph nodes and in distant organs where lymphatic vessels are located, such as the lungs (11, 45). In this model, it is assumed that endometrial cells can pass into the lymphatic and vascular circulation without interruption and then undergo extravasation to be transplanted within distant organs (43). However, there is no scientific evidence to support the theory that menstrual tissue arising from benign endometriosis can perform these difficult cancer-like tasks (43, 46).

### Stem cell recruitment theory 

This theory may be supported by the periodic renewal of the endometrium after the shedding of the superficial layers that occurs through the proliferation of the remaining basal layer of the endometrium. This layer, which does not fall off and remains to rebuild the endometrium, is supposed to contain stem cells (12). The stem cell population represented by clonogenic cells has been identified and their involvement in endometriotic lesions has been suggested (47). In the case of endometriosis, there are 2 main alternatives based on stem cell origins. The first origin is stem cells of the basal layer of endometrium while the second is the bone marrow. Regardless of the origin of the stem cells, other factors are postulated to be involved in the DOE. These include hormones and other molecular factors present in the tissue microenvironment (43). Endometrial stem cells are located in the proximal ends of the basal glands at the interface between the endometrium and the myometrium. They rebuild the superficial layers of the endometrium when stimulated by estrogen (48). Stem cells from the endometrium are supposed to pass outside the uterus during RM and cause endometriosis (43). On the other hand, the bone marrow origin of some endometrial stem cells may explain the hematogenous spread of distant endometriotic lesions (12).

### Immune dysfunction theory 

The high prevalence of autoimmune disease in patients with endometriosis may support the possibility of immune dysfunction in the pathogenesis of endometriotic lesions (49). It has been reported that decreased cellular immunity, increased activated macrophages, and suppressed natural killer cell function are observed in patients with endometriosis (50). There may be a relationship between immune dysfunction and the DOE. Immune cells, including macrophages, neutrophils, natural killer cells, and dendritic cells, can play a role in the pathogenesis of endometriosis through angiogenesis as well as the growth and invasion of endometrial cells. These immune cells secrete defensins and cytokines that also influence the environment surrounding the DOE (51). However, these immune dysfunctions may be a tissue reaction in response to the development of ectopic endometriosis.

### Hormones' imbalance theory 

Endometriosis is a disease that is highly dependent on estrogen. Hormonal treatment of the disease is mainly based on reducing the endogenous ovarian production of estrogen (52). Therefore, the hormone plays a crucial role in the DOE. The evidence for this is that the disorders affect women of reproductive age and improve after menopause. Furthermore, environmental toxins that mimic estrogen such as dioxin are involved in the etiology of endometriosis (12). There is also a high bioavailability of estradiol in endometriotic lesions. This may be due to decreased conversion of estradiol to the less potent estrone due to decreased expression of the 17 β -hydroxysteroid enzyme in ectopic endometrial tissue (38). On the other hand, progesterone can inhibit the action of estrogen. An imbalance in the regulation of these 2 hormones such as estrogen dominance and progesterone resistance can lead to the DOE (53). Progesterone resistance may be due to decreased expression of progesterone receptors in the ectopic endometrium (54). Hormonal imbalance can also be caused by decreased levels of micro ribonucleic acids: 23a and 23b and an increase in micro ribonucleic acids: 135a and b, 29c and 194-p (55).

### Environmental theory 

Environmental factors have an impact on the risk of DOE (56). These include lifestyle and organic pollutants. A sedentary life and lack of physical activity, as well as increased intake of alcohol, smoking, and caffeine lead to a malfunction of the human body, which increases the risk of endometriosis. Physical activity is supposed to normalize the level of estrogen and regulate menstrual flow. Furthermore, smoking can disrupt the synthesis of natural steroids and prostaglandin E2. Alcohol also opposes hormone synthesis compared to caffeine (11, 56). On the other hand, organic pollutants including dioxins and polychlorinated biphenyls produced in industrial processes can disturb the endocrine system and may accumulate in adipose tissue in cases of deep infiltrating endometriosis (11, 56–58). In addition, the presence of industrial air pollution is associated with increased oxidative stress in humans (59). Some authors have emphasized the role of oxidative stress in the pathophysiology of endometriosis leading to an inflammatory peritoneal cavity response (60).

## 6. Ciliary beats and muscle contraction in the fallopian tube

The fallopian tubes are a pair of hollow muscular tubes that connect the uterine cavity to the pelvic peritoneal cavity in the ovary region. The tubular mucosa shows longitudinal folds and is lined by 2 main cells, ciliated and secretory cells. Ciliated cells appear mostly at the apex of the mucosal folds. It increases gradually from the isthmus, where it constitutes less than 35% of the cells, toward the distal end of the tube at the fimbria, forming more than 50% of the lining cells (61). Cilia aid in the movement of tubal fluid and its contents toward the uterine cavity (62). In humans, the fallopian tube plays a crucial role in fertility as it is the site of fertilization and is also involved in the movements of the fertilized ovum or developing embryo toward the uterine cavity. Moreover, its open lumen helps transport sperms to the site of fertilization in the lateral third of the tube. Rhythmic muscle contractions, called peristalsis, and the swaying movements of the cilia of the fallopian tube wall work together to move the mature egg, whether fertilized or not, to reach the uterine cavity within 3–4 days (6, 63). The secretions of the mucous cells in the fallopian tube, rich in glycoproteins and growth factors, aid fertilization and embryo development (63, 64). Therefore, the tubal transit of the developing fetus into the uterine cavity depends mainly on 3 factors. The first factor is the beating of the lining cilia, the second is a contraction of tubular muscles, and finally, the third factor is tubular epithelial secretions (65). Female steroid hormones, including estrogen and progesterone, can modify the function of fallopian tube cells. The estrogen can increase tubal fluid secretion and ciliary beat frequency (62). The frequency of the ciliary beat of the tube also increases at ovulation and in the secretory phase of the uterine cycle, which helps transport and empty the contents of the tube toward the uterine cavity (64). When the ciliary movement of the tube is disrupted due to extensive trauma to the tubal epithelium, such as in cases of pelvic inflammatory disease or sexually transmitted diseases, fertility can be affected. Sluggish or disturbed ciliary movements may also lead to an ectopic pregnancy if fertilization occurs (63, 66, 67).

## 7. Female SADM

Previous studies regarding women's SADM and its relationship to the emergence and DOE, appear to be few worldwide. This may be because there is no convincing explanation as to whether these sexual activities during that period could have caused this challenging disease (Table II). A study conducted in Philadelphia, USA, reported that intercourse during menstruation increases the risk of endometriosis (26). Unlike the previous study, study from Yale University found no association (68). Instead, the researchers suggested that sexual activities during menstruation could provide women with protection from endometriosis. However, the latest study relied on mailed surveys for the control group, without relying on laparoscopy or laparotomy for women not affiliated with the organization. On the other hand, recent reports in Iran have confirmed the association between orgasms resulting from female sexual activities during menstruation and the DOE (4, 69). These reports are consistent with another study in Qatar that confirmed the association of these sexual practices during menstruation with the appearance of endometriosis (70). However, this study relied only on ultrasound and magnetic resonance imaging for diagnosis. Although these imaging techniques are suggested, they are not the definitive criterion for the diagnosis of endometriosis as this is mainly based on laparoscopy or surgery with histopathological verification of endometrial tissue (71).

**Table 2:** Some studies regarding the relationship between female sexual activities and the DOE


**Authors, Yr (Ref)**	**Country**	**Methods**	**Conclusions**
**Filer and Wu, 1989 (26)**	USA	Laparoscopy or laparotomy	Sexual intercourse during menses can increase the incidence of endometriosis
**Meaddough ** ***et al.*** **, 2002 (68)**	USA	Mailed surveys	Sexual activity accompanied by orgasm during menstruation may lead to protection against endometriosis
**Samir ** ***et al*** **., 2011 (70)**	Qatar	Ultrasonography and MRI	Sexual intercourse during menstruation can lead to endometriosis
**Mollazadeh ** ***et al*** **., 2019 (4)**	Iran	Laparoscopy and laparotomy with histopathological diagnosis	Sexual activity accompanied by orgasm during menstruation is associated with endometriosis
All the study type was case-control study. DOE: Development of endometriosis, MRI: Magnetic resonance imaging			

## 8. Female orgasm (FO)

Orgasm, which is a pleasurable series of perineal and vaginal muscle contractions, can also be brought about by female masturbation through physical stimulation of the clitoris, vulva, and vagina, or called clitourethrovaginal complex (72). Uterine contractions that occur during orgasm are supposed to help upsuck semen that deposits in the vagina during intercourse (73, 74). Although the upsuck hypothesis is not primarily correlated to the number of offspring and even the occurrence of fertilization itself (75), it can help push sperm through the uterus and into the fallopian tube (76). Orgasm can be initiated as a result of sexual arousal by sexual intercourse, and/or sexual manipulation of the female's external genitalia and perineal region (77). It has been stated that SADM associated with orgasm, whether via vaginal intercourse or sexual arousal, is a critical factor in the DOE (4). The unusual retrograde motion of endometrial tissue through the fallopian tubes occurs in the opposite direction to the normal flow of fallopian contents that helps the developing zygote reach the uterine cavity (78). This unusual flow of the fallopian tubes may only occur with orgasm to help push sperms, which are swimming upstream, to reach the site of fertilization in the distal part of the fallopian tubes (6).

On the other hand, estrous animals do not show spontaneous endometriosis. However, it can happen by induction through transplantation of parts of the endometrium either from the same animal called a homozygous model or from a human to an immunocompromised animal called the heterozygous model (79). Female animals are, usually, only sexually active during estrus or the second phase of the cycle called “in heat". In contrast, females of a menstruating species can be sexually active at any time in the cycle, even when they are menstruating (80). However, women tend to have more sexual thoughts and motivation and are more likely to engage in sexual activity around the time of or just before ovulation (81).

Many women report an orgasm from some form of sexual arousal other than intercourse (82). FO can be induced by sexual stimulation of the genitals and some non-genital sites. It is characterized by involuntary and rhythmic contractions of the pelvic muscles around the vagina, with accompanying uterine and anal contractions resolving vascular congestion and muscle tension caused by sex, generally with stimulation of a state of well-being and satisfaction (77).

The orgasm stimulation of females is primarily achieved through physical manipulation of the genitalia or sometimes from vibratory mechanical devices. Although activities that induce FO usually focus on the genitals, FO may occur through stimulation of other parts of the female body (83).

A woman's drive to orgasm may vary with different sexual behaviors. Some women are more likely to orgasm in behaviors that involve clitoral stimulation, rather than just vaginal penetration (84). Furthermore, other women experience an orgasm when exposed to other sexual behaviors such as foreplay, manual stimulation of the genitalia, and oral sex. While about 25% of females report reaching orgasm through sexual intercourse, the largest proportion (about 32%) of women experience orgasm with oral sex (85).

The anal region is very sensitive to stimulation. The anal region contains a dense network of sensory nerves that are involved in the engorgement and contractions of the genitals leading to sexual arousal and FO. Therefore, touching/stimulating the anus can lead to an intense orgasm in some women (86). Because of the close relations between the anus and the anal canal with the vaginal wall, anal intercourse may indirectly stimulate the erogenous zones in the anterior wall of the vagina resulting in the creation of a powerful FO (87). This is in addition to stimulating the sensory pudendal nerve endings distributed in the perineal region. The pudendal nerve “S2-S4", which arises from the sacral plexus, transmits sensory, motor, and autonomic inputs from the perineal region including the perineal muscles, genitalia, and anus (88). Furthermore, as many as 40% of women feel the anus as a pleasant area of sexual touch coinciding with vaginal intercourse or clitoral stimulation (86).

## 9. Conclusion

It has been concluded that endometriosis is a painful and debilitating disease that is often associated with infertility and negatively affects all aspects of a woman's life. Moreover, its treatment remains a medical challenge; therefore, avoiding its occurrence is a top priority. RM remains the most compatible etiological theory for endometriosis. The onset of FO during menstruation can be a possible cause of such RM, leading to endometriosis; however, its exact causes are still unclear. The sexual activities that a woman engages in during this period may indicate the risk of endometriosis resulting from the upsuck mechanism of the reproductive ducts that can occur as a result of FO. Future studies are highly recommended to substantiate this hypothetical explanation and to further clarify all aspects of endometriosis. This can help avoid endometriosis, which is a major medical challenge.

##  Data availability

Research data associated with the paper is available, and further information can be accessed from the author upon reasonable request.

##  Author contributions

The author had full access to all of the data in the study and takes responsibility for the integrity of the data and the accuracy of the data analysis.

##  Conflict of Interest 

The author declares that there is no conflict of interest.
